# Leveraging Open-Source Large Language Models for Data Augmentation in Hospital Staff Surveys: Mixed Methods Study

**DOI:** 10.2196/51433

**Published:** 2024-11-19

**Authors:** Carl Ehrett, Sudeep Hegde, Kwame Andre, Dixizi Liu, Timothy Wilson

**Affiliations:** 1 Watt Family Innovation Center, Clemson University, Clemson, SC, United States; 2 Department of Industrial Engineering, Clemson University, Clemson, SC, United States; 3 Department of Computer Science, Clemson University, Clemson, SC, United States

**Keywords:** data augmentation, large language models, medical education, natural language processing, data security, ethics, AI, artificial intelligence, data privacy, medical staff

## Abstract

**Background:**

Generative large language models (LLMs) have the potential to revolutionize medical education by generating tailored learning materials, enhancing teaching efficiency, and improving learner engagement. However, the application of LLMs in health care settings, particularly for augmenting small datasets in text classification tasks, remains underexplored, particularly for cost- and privacy-conscious applications that do not permit the use of third-party services such as OpenAI’s ChatGPT.

**Objective:**

This study aims to explore the use of open-source LLMs, such as Large Language Model Meta AI (LLaMA) and Alpaca models, for data augmentation in a specific text classification task related to hospital staff surveys.

**Methods:**

The surveys were designed to elicit narratives of everyday adaptation by frontline radiology staff during the initial phase of the COVID-19 pandemic. A 2-step process of data augmentation and text classification was conducted. The study generated synthetic data similar to the survey reports using 4 generative LLMs for data augmentation. A different set of 3 classifier LLMs was then used to classify the augmented text for thematic categories. The study evaluated performance on the classification task.

**Results:**

The overall best-performing combination of LLMs, temperature, classifier, and number of synthetic data cases is via augmentation with LLaMA 7B at temperature 0.7 with 100 augments, using Robustly Optimized BERT Pretraining Approach (RoBERTa) for the classification task, achieving an average area under the receiver operating characteristic (AUC) curve of 0.87 (SD 0.02; ie, 1 SD). The results demonstrate that open-source LLMs can enhance text classifiers’ performance for small datasets in health care contexts, providing promising pathways for improving medical education processes and patient care practices.

**Conclusions:**

The study demonstrates the value of data augmentation with open-source LLMs, highlights the importance of privacy and ethical considerations when using LLMs, and suggests future directions for research in this field.

## Introduction

### Overview

Generative large language models (LLMs) are powerful technologies that leverage machine learning techniques to generate novel and contextually relevant content. By training on vast amounts of data, LLMs have the capability to understand and mimic human language patterns, thereby producing text that closely resembles human-written content [1,2]. LLMs represent a subset of generative models characterized by their vast training data and resulting complexity. With billions of parameters, LLMs such as GPT-3 and GPT-4 by OpenAI are capable of generating text that is often indistinguishable from human-written content, provided a suitable context is given (OpenAI) [3].

The use of LLMs has the potential to address critical challenges in medical education. In environments where teaching resources are limited, these models can generate learning materials from case studies to interactive dialogues that align with specific learning objectives and target specific topics [4,5]. Furthermore, they can create diverse and complex patient scenarios that can supplement lecture content by providing real-time clarifications, and context to complex topics, ensuring a deeper understanding for students [6]. By leveraging the capabilities of LLMs, educators can identify content gaps, ensure comprehensive coverage of essential subjects, and ultimately enhance the quality and effectiveness of medical education [7,8]. These models can enhance teaching efficiency and learner engagement, thereby potentially improving learning outcomes.

LLMs, however, pose several challenges in their application in medical education. Ethical use and privacy concerns need to be considered, especially when using real-world data for training. Cost concerns might arise due to the computational resources needed for training and fine-tuning these models. GPT-3 and GPT-4 (and the product ChatGPT which is built upon them) are closed-source models owned by OpenAI; their use thus not only comes at a financial cost, but also generates privacy concerns due to needing to expose one’s data to a third-party company. While open-source LLMs exist, relatively little attention has been paid to their utility, despite the fact that they alleviate both cost and privacy concerns attached to the use of commercial LLMs.

The application of these models in medical education is becoming increasingly prevalent. For instance, LLMs have been used to aid revolutionizing medical curriculum development [8,9], teaching methodologies [10], personalized study plans and learning materials [11], assessments and evaluation [12,13], medical writing and assistance [14,15], and medical research and literature review [16,17]. The vast potential of these technologies opens up novel avenues for educating the future generation of health care providers.

Over the past few decades, self-reported data from health care workers, such as incident reports, have been applied to medical education in many health care areas. These include analyzing potential ethical conflicts within hospitals [18], evaluating Bendamustine-related skin disorders [19], finding predictive patterns of human contributing factors in radiation therapy [20], and improving patient safety and care [21-23]. Despite these applications, LLMs have so far, not been used in the analysis of self-reports. There is an opportunity to leverage hospital self-reports to enhance medical education.

Integrating artificial intelligence (AI) and LLMs in self-report analysis has the potential to revolutionize bottom-up learning from worker-generated data, facilitating more efficient and accurate identification of workflow challenges, systemic issues, strategies and tactics to address these, and areas for improvement in clinical decision-making and patient care. This study addresses the use of LLMs, particularly open-source LLMs, to mitigate a specific problem encountered in the analysis of hospital staff survey data: the lack of ample training data for a text classification task. This task involves classifying text responses into categories based on their relevance to the availability of resources in the hospital. Insufficient training data can limit the model’s ability to learn and make accurate classifications.

The objective of this study is to evaluate the effectiveness of using open-source LLMs for data augmentation in this text classification task. By generating synthetic survey responses, LLMs can potentially increase the size and diversity of the training dataset, leading to improved model performance in text classification. Text classification, in turn, is a useful way to analyze free-text reports for categories and themes that are relevant from an educational standpoint. In our research, text classification is used to identify valuable insights from self-reported narratives of the lived experiences of frontline health care workers. Identifying such patterns and capabilities that are situated in the context of everyday work, can be valuable in generating teachable content for medical education. Doing so with augmented data would allow for a richer dataset of realistic learning instances based on everyday work. This paper presents a case study of this approach, aiming to provide insights and guidance for similar applications in medical education and health care operations.

### Related Work

Data augmentation is the process of generating new data from existing data. This process is generally used to increase small datasets or create more diversity in a dataset where underrepresented populations are ignored by the model. A lack of diversity in a language model’s training set can lead to poor generalizability. For example, LLMs performing numerical reasoning perform better on tasks with terms seen frequently during training, with a gap in accuracy of up to 70% when solving problems containing terms common in the training data as opposed to rare terms [9]. Few-shot learning is generally used with data augmentation because of the lack of usable data and its ability to be efficient with small amounts of data. LLMs are either used to slightly change examples to create new data or generate new data from examples. Using methods that change specific words in slot filling fill in the blank [24], where those words are switched with a semantically similar word, is a widely used method to change existing data slightly. Generative models typically use fine-tuned versions of LLMs [25,26] with prompts, including select examples from the dataset and the label the model is supposed to generate. Zero-shot prompting has also been used with ChatGPT [27,28] in low-resource situations. Models that have received no fine-tuning have also been shown to perform well [29] train an intent classifier, and feed it into the LLM to generate data. Human-in-the-loop studies have been shown to be successful. A human expert filters through generated data and discards generated data that deviate from the training data [30]. Another filtering technique is using a binary sentence classifier to determine whether the original and the augment are semantically similar. We expand the existing literature in this space by exploring the case of low-resource data augmentation in the face of cost and privacy concerns that prevent relying on third-party services such as OpenAI’s ChatGPT, using few-shot prompting on open-source LLMs.

## Methods

### Ethical Considerations

This research study does not require institutional review board approval according to Clemson guidelines [31]. The project involves analysis of a preexisting anonymized dataset, and thus does not constitute research involving human participants as outlined in the federal regulations [45 CFR 46.102(e)]. Our research involves neither obtaining information through intervention or interaction with living individuals, nor the use, study, analysis, or generation of identifiable private information. This type of secondary data analysis, where the researchers do not have access to identifying information, is not considered research involving human participants and therefore does not require institutional review board oversight.

### LLMs for Data Augmentation

We use LLMs for data augmentation, specifically focusing on Large Language Model Meta AI (LLaMA) and Alpaca models. LLaMA is a collection of foundation language generation models with varying complexities ranging from 7 billion (7B) to 65 billion (65B) parameters, introduced by [10]. These models were trained on approximately 1.4 trillion (1.4T) tokens, an extensive dataset derived entirely from publicly accessible sources, thus eliminating dependency on proprietary databases and increasing transparency. The models themselves are open-source and freely available to researchers.

In terms of their architecture, LLaMA models are built on the transformer architecture [32] and incorporate several advancements proposed in recent research, including prenormalization [33] for improved training stability, the SwiGLU activation function [34] for enhanced performance, and rotary embeddings [35] for improved positional encoding. Notably, even at a comparatively smaller scale, LLaMA models are competitive with GPT-3 (175B) in a wide variety of benchmarks. Their combination of small size (and corresponding computational accessibility) with competitive performance, in conjunction with their status as open-source, motivated our choice to focus our work on these models.

Alongside the LLaMA models, we use a set of Alpaca models in our experiments. These models are LLaMA models that have been fine-tuned by Taori et al [36] for instruction-following tasks using a 52K dataset consisting of instructions and corresponding text responses. We include Alpaca models in order to investigate whether this instruction fine-tuning step might make the models more adept at data augmentation tasks. All of the models used in our study were sourced from Huggingface’s library.

### LLMs for Classification

Robustly Optimized BERT Pretraining Approach (RoBERTa), XLNet, and DistilBERT (Distilled BERT) are all LLMs that have been pretrained on a large corpus of text data. They can be fine-tuned for a variety of tasks, including text classification, natural language inference, and question answering.

RoBERTa stands for “Robustly Optimized BERT Pretraining Approach” [37]. It is a BERT-based model that has been trained on a larger corpus of text and with more training steps than the original BERT model, with a modified training objective. This makes RoBERTa more accurate than the original BERT on a variety of tasks.

XLNet [38] is a transformer-based model that has been trained on a corpus of text that has been masked and shuffled. This makes XLNet more robust to noise and errors in the training data than other LLMs. While both XLNet and RoBERTa are transformer-based language models, the key difference is in their training methods. RoBERTa is a variant of BERT using dynamic masking and longer training on larger amounts of data. In contrast, XLNet uses a permutation-based training approach where all permutations of words in a sentence are considered during prediction, with the goal of providing a more comprehensive contextual understanding.

DistilBERT [39] is a smaller version of BERT that has been trained to have the same performance as BERT on a variety of tasks. DistilBERT is faster and uses less memory than BERT, making it a more practical choice for many applications due to its smaller size.

In this paper, we use RoBERTa, XLNet, and DistilBERT as text classifiers to test the effectiveness of our data augmentation. For each generative LLM used for data augmentation, we use synthetic data to supplement our real survey responses. Thus, after augmentation, we have a larger dataset of text documents, each of which is associated with a label: “resource” or “nonresource” related. The real survey responses are manually labeled by us. The synthetic data cases are (written and) labeled by the LLM. This larger dataset is then used to fine-tune each of the 3 classification models on the task of determining, for a given piece of text, whether it is “resource” or “nonresource” related.

We gather augments (synthetically generated text responses) with different models, temperatures, and training sets. We chose a binary classification scheme of classifying sentences as either “resource”-related, or “non-resource”-related. This choice was based on the fact that a substantial proportion of resilience engineering tools to improve patient safety (RETIPS) reports were found to be related to resources, including the availability of necessary resources, such as staff and equipment. Each time an augment is generated, a new few-shot learning prompt is generated by randomly sampling and concatenating 5 examples each of resource-related and nonresource-related survey responses from our (real) labeled data, displayed in the format shown in Textbox 1.

Textbox 1. **Prompt template used for few-shot prompting to generate synthetic data. “Category” could be either “resource-related” or “nonresource-related,” depending on which type of data the model is intended to generate.**

### Instruction:

Here are two lists of short text documents, \

“Resource-related“ and “Nonresource-related”. \

They are survey responses by hospital staff \

at the Children’s Hospital of Philadelphia (CHOP).

“Resource-related” is responses on the topic \

“Availability of resources OR Knowing where to find resources.”

“Nonresource-related” is responses that do not have to do with that topic.

Please give me a new example of a short text document that would belong \

in the “{category}” category.

Please don’t copy or paraphrase the text documents in the \

input lists I give you; instead, come up with your own new example \

that would belong in the “{category}” list.

### Input:

{other_category}:{other_category_examples}

{category}:{category_examples}

### Response:

{category}:

1.

After the synthetic data have been generated, it is filtered to retain only those model outputs that include alphabetic characters, since in some cases the model returns an empty output or simply a continuation of the numerical list begun by the “1.” at the end of the prompt (eg, “2. 3. 4. 5.”) These problems were especially common at low temperatures (0.1 and 0.3), leading us to exclude those temperatures from further consideration. For each tested combination of text-generating LLM, classifier LLM, and temperature, the filtered augments from the text-generating LLM at that temperature are combined with the real labeled data and used to fine-tune the classifier LLM, which is then evaluated on a hold-out set of real labeled data. Examples of generated data are shown in Table 1.

**Table 1.** Examples of real data and synthetic data from each text-generating large language model.

Model	Responses
Real data	How quickly the world changed...I remember the work and practice pre-COVID and can’t believe how quickly we went to working remotely and virtually. I was also surprised at how adaptable we really are...we think people are resistant to change but people can change incredibly swiftly wen the world changes and they need to adapt. I was also surprised to learn that there are so many online telemeeting platforms...it is routine to oscillate between 3‐4 programs in sequential meetings. I believe overall we did exceptionally well. PPE^a^ availability and consistency across the enterprise would be an area I think we could improve upon. usage of ppe Virtual conferencing improved our conference participation and virtual review of imaging with referring clinicians using BlueJeans business Skype or FaceTime improved patient Care and physician collaboration. Unfortunately the hospital doesn’t have business Skype installed on hospital computers just on lab tops. There needs to be a uniform hospital wide platform. Working remotely required an adjustment in how I communicated about everything. The demands of the pandemic required an adjustment in priorities which put everything on hold except for COVID related work and it required a level of teamwork that did not exist pre-COVID. From a personal coping standpoint I had to “schedule” time away from my computer each day to eat lunch and take a walk.
LLaMA-7B^b^	I appreciate the ability to work from home and the support that was provided from my department. I did miss the interaction with coworkers but the ability to work from home was worth it. I was able to keep up with my work and the department was able to I am not aware of the term “telemeeting platforms” but I believe I could answer this question. I am not sure if I was surprised by the need to adapt or the speed at which we adapted. I think we were all expecting the need for changes and adapt It was a challenging time for our department as we did not have enough PPE for everyone and we had to ration supplies for the most essential staff. We were able to support our staff and be very open and fluid with how things were evolving. The ability to go to the bathroom and not have to worry about getting someone else sick Changes in Policies and procedures having all the PPE needed communication with all staff AWA leadership was helpful and tranparent
LLaMA-30B	In the beginning working AWA^c^ was an adjustment but once I was allowed to bring home my docking station and 2 monitors it was just like working in the office with little or no interruptions. My doctors I supported and I kept in constant contact through phone call We had a lot of staff on site so we could provide a good flow of patients and care. In the beginning working AWA was an adjustment but once I was allowed to bring home my docking station and 2 monitors it was just like working in the office with little or no interruptions. I am confident that I can enter the building safely and that I can maintain the safety of staff, patients and families. Constant communications with the changes is helpful. It is also good that I can look in one place to refresh on any of the topics. I was surprised how quickly we went to working remotely and virtually.
Alpaca-7B	I was surprised at how quickly the world changed from in-person interactions to virtual ones. I also didn’t realize how adaptable people could be in terms of adjusting to the new way of working. I was also surprised to learn about the different online telemeeting platforms that are Not having to share Ascom phones and not having an office adjacent to patient care areas. Followed protocols put in place and used PPE and hand sanitizer and bought my own when I felt uncomfortable with there maybe not being enough or “the right kind” for me. How quickly the world changed...I remember the work and practice pre-COVID and can’t believe how quickly we went to working remotely and virtually. The hospital’s ability to partner with other departments to obtain the supplies and resources needed to ensure AWA roles were fully functional.
Alpaca-30B	As a manager, I am grateful for the availability of resources in the workplace. We have a well-stocked supply of PPE and other equipment that we need to do our jobs effectively. Additionally, we have access to a wide range of educational materials and training opportunities. The availability of clinical information and resources were limited during the pandemic, but we were able to navigate through this challenge by establishing a robust telemedicine platform that allowed for remote consultation and collaboration with our colleagues. Ensuring that all staff members are aware of the available resources and are able to easily access them is crucial for maintaining patient safety and reducing stress levels in the workplace. The biggest adjustment for me was how to handle the stress of working with a reduced staff and how to prioritize my workload. The CHOP^d^ website and intranet were helpful in providing information about the availability of resources and where to find them.

a PPE: personal protective equipment.

b LLaMA: Large Language Model Meta AI.

c AWA: alternative work arrangements.

d CHOP: Children’s Hospital of Philadelphia.

**Figure 1.**
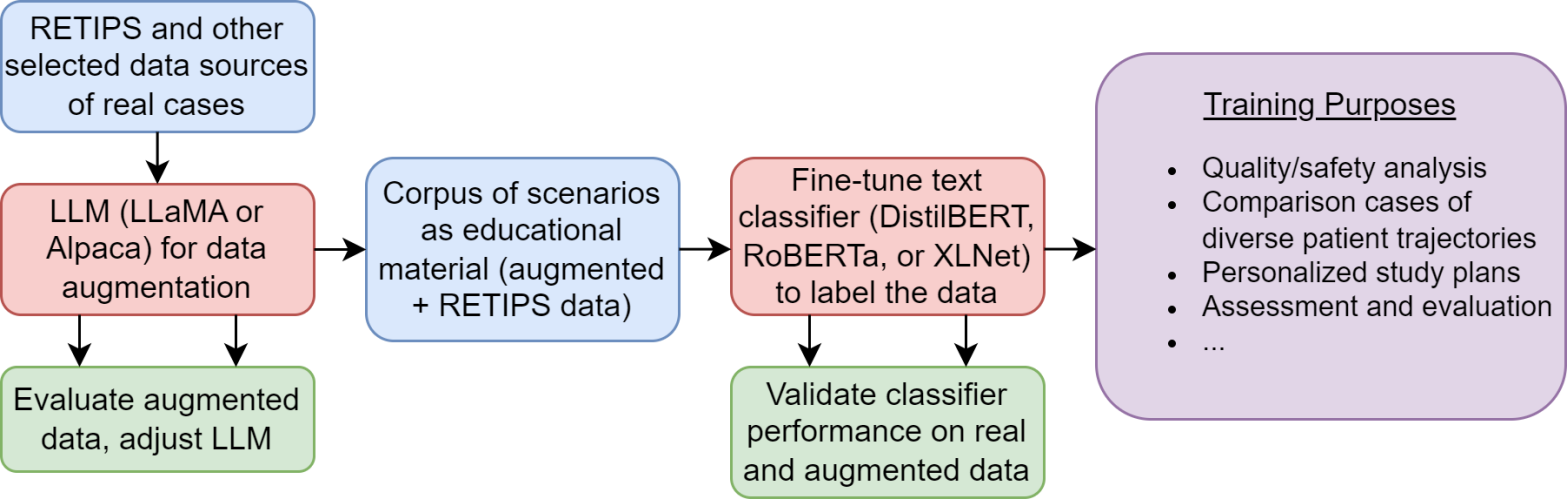
A framework for long-term implementation of LLMs for medical education using RETIPS and similar self-reported data. DistilBERT: Distilled BERT; LLaMA: Large Language Model Meta AI; LLM: large language model; RETIPS: resilience engineering tool to improve patient safety; RoBERTa: Robustly Optimized BERT Pretraining Approach.

Our methodology fits within an envisioned framework represented in Figure 1. Using data from RETIPS and similar self-reported narratives from frontline staff, LLMs with data augmentation will result in a corpus of scenarios. Human stakeholders (eg, educators) can interact with LLMs to compare models, change temperature, and make other adjustments, based on the results, in an iterative manner until the quality and representativeness of augmented data are deemed satisfactory. These scenarios can be used for a variety of purposes in medical education, including quality and safety analysis, creating content for personalized study, and more. Based on their effectiveness in the training, including student and instructor feedback, the LLMs can be further fine-tuned for improvements. The work described here represents a part of this framework, focusing on the development and evaluation of LLMs for data augmentation.

## Results

We analyze the performance of 4 distinct LLMs—LLaMA-7B, LLaMA-30B, Alpaca-7B, and Alpaca-30B—for the purpose of data augmentation. The goal of the augmentation was to increase the performance of downstream classifiers on the task of matching human labelers’ categorization of the text data as “resource” or “nonresource” related. We thus evaluate the quality of the resulting data augmentation by adding the synthetic data from these LLMs to the data used to train 3 classifiers: DistilBERT, RoBERTa, and XLNet. We repeat this analysis for 6 different augmentation “temperature” settings ranging from 0.5 to 1.5. The performance of each model-classifier-temperature combination is assessed based on the area under the receiver operating characteristic (AUC) curve, using a holdout set of human-labeled data.

The overall best-performing combination of LLM, temperature, classifier, and number of augments is LLaMA 7B at temperature 0.7 using RoBERTa with 100 augments, with an average AUC of 0.87 (SD 0.02: 1). In addition to achieving the highest absolute performance, the data augmentation is also most beneficial in this case—this augmentation yields the greatest improvement in AUC with respect to the baseline performance of that classifier model with no data augmentation. The baseline performance of each classifier along with optimal data augmentation for each text-generating LLM is shown in Table 2. Note that the fine-tuned Alpaca models do not outperform the LLaMA models upon which they are based, indicating that instruction-finetuning is not necessary for this data augmentation task.

**Table 2.** Comparison of classifier performance under augmentation by each text-generating large language model (LLM), alongside base performance of the classifier with no augmentation. Each entry gives the mean classifier area under the receiver operating characteristic curve (SD 1), the optimal temperature for text generation.

LLM	RoBERTa^a^, mean (SD)	XLNet, mean (SD)	DistilBERT^b^, mean (SD)
LLaMA-7B	0.87 (0.02/0.7/100)	0.84 (0.04/0.7/100)	0.83 (0.02/0.7/100)
LLaMA-30B	0.87 (0.03/0.5/100)	0.84 (0.03/0.5/100)	0.85 (0.06/1.5/500)
Alpaca-30B	0.86 (0.06/0.7/100)	0.84 (0.05/1.3/250)	0.81 (0.06/1.3/250)
Alpaca-7B	0.86 (0.06/0.7/100)	0.84 (0.05/1.1/250)	0.82 (0.05/0.7/100)
Baseline	0.80 (0.06)	0.79 (0.06)	0.79 (0.04)

a RoBERTa: Robustly Optimized BERT Pretraining Approach.

b DistilBERT: Distilled BERT.

**Figure 2.**
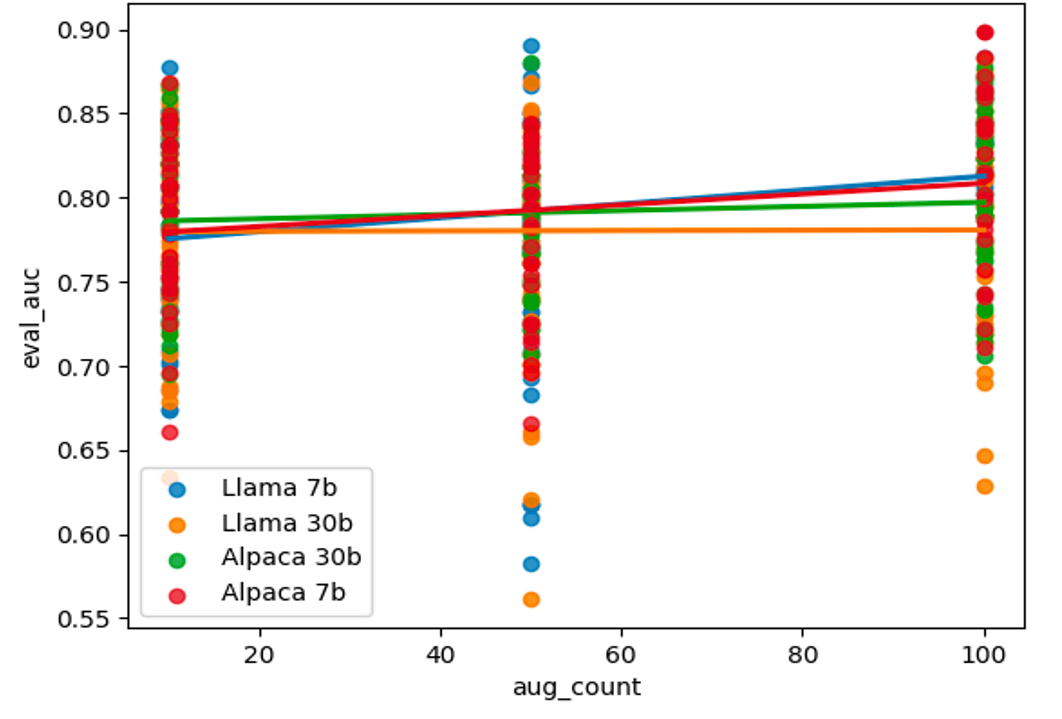
Linear fit to DistilBERT (Distilled BERT) model performance (measured as area under the receiver operating characteristic curve) as a function of the number of augments included in the training data (all generated at temperature 0.7).

For each combination of LLM and classifier, we also fit a linear regression model to explore the relationship between the number of synthetic data points included in the training dataset and the resulting classifier performance as measured by AUC. Notably, DistilBERT emerges as the classifier benefitting most often from data augmentation. In terms of the temperature setting, most of the successful models used a temperature of 0.7. In Figure 2, we display the comparative performance of the LLaMA 7B and Alpaca 7B models, both using the DistilBERT classifier at a temperature setting of 0.7. These graphical representations underscore the beneficial impact of data augmentation on the AUC performance of these specific model-classifier configurations.

## Discussion

### Principal Findings

In this work, our emphasis is on leveraging open-source language models that strike a balance between computational performance and accessibility for researchers. We therefore set a parameter ceiling of 30 billion parameters for several reasons. First, maintaining this threshold ensures that the models in question can run on consumer-level hardware commonly available to average researchers without the need for prohibitive investment in specialized equipment. Second, this approach aligns with our goal to propose methods feasible for environments where privacy and cost considerations limit the use of third-party cloud-based computing services, as relying on external infrastructures (such as OpenAI’s services) could elevate privacy risks and regulatory complexity. Using consumer-level hardware, as opposed to cloud-based services, significantly mitigates the risk of data breaches or unauthorized access. Furthermore, the choice to avoid third-party computational services also avoids potential issues related to data sovereignty and control, which could arise when data leaves the institution’s local environment. By strictly using in-house resources that operate within the confines of consumer-level capabilities, our methodology facilitates stringent data custody and integrity controls.

LLMs such as OpenAI’s GPT-3.5 and GPT-4, or the openly accessible 176 billion-parameter BLOOM, indeed offer more powerful capabilities, but their deployment would threaten the objective of presenting a methodology that is both privacy-aware and broadly implementable. We contend that models up to 30 billion parameters offer a sweet spot, considering these constraints, without significantly compromising the efficacy of the data augmentation process. By imposing a limit of 30 billion parameters, we aim to demonstrate that effective data augmentation for small-scale text classification tasks in the health care sector can be achieved without resorting to the most computationally demanding or privacy-compromising technology. This parameter threshold also allows for an equitable comparison of language models, ensuring that our results are relevant to a wide range of researchers, including those who might be limited by resource constraints. Our research thus serves to bridge the gap typically present in medical informatics research, where smaller institutions or individual researchers may not have access to the same level of computational resources as their larger counterparts. Additionally, this study sheds light on the possibilities and limitations inherent to working within such constraints, providing a valuable reference for future research endeavors seeking a similar balance between model size, privacy, cost, and performance.

### Limitations and Future Work

One of the primary constraints of this work is the limited size of the RETIPS dataset. The small sample size (58 responses) potentially affects the reliability and generalizability of the study to other cases where larger data are available. However, it should be noted that the data from RETIPS were tightly focused on a narrow set of themes. This may be beneficial to the quality of augmented data, when compared with a dataset that is thematically more “scattered” or heterogenous. Larger data would likely improve the data augmentation quality but would potentially limit the benefits to be derived from data augmentation. Since data augmentation is of greatest value when working with small datasets, our small data size helps explore this problem space.

Aside from data size, another limitation of this work is that our data are exclusively collected from RETIPS surveys administered to radiology staff at a single hospital. Though this specificity is necessary for the research’s objectives, the models’ performance may vary in other health care domains, and in domains outside of medicine.

As a future step for this research, it would be beneficial to perform similar studies using larger and more diverse datasets. Larger datasets could provide a richer, more diverse range of training data, potentially leading to correspondingly more diverse synthetic data. Such diversity could improve the performance of the downstream classifiers.

Future research should also consider experimenting with different LLMs for data augmentation. Text-generation LLMs are rapidly evolving, particularly in the area of making large and powerful LLMs accessible on consumer-grade computer hardware [40]. Newer models often come with architectural and training improvements that could potentially enhance synthetic data generation quality.

In this work, we explore different LLM text generation temperature settings and the resulting impact on synthetic data quality. A more extensive hyperparameter tuning of the language models and classifiers may yield further improvements in their performance. This could be a fruitful area for further investigation.

The limitations faced by this work are those that inherently attach to working with small data in a highly privacy-conscious environment with accessible AI tools. Since this is a problem space occupied by many researchers and practitioners in the health care domain, we hope that our results are able to provide insight into how AI tools can be used in such settings.

### Conclusion

This study provides an exploration and practical demonstration of the application of LLMs for data augmentation in the context of health care. We specifically focused on the use of open-source LLMs, namely, LLaMA and Alpaca models, to mitigate the challenge of limited training data in a text classification task related to hospital staff surveys.

Our findings demonstrate the potential effectiveness of using LLMs to generate synthetic survey responses, thereby increasing the diversity and size of the training dataset and improving the performance of models trained on the augmented dataset for tasks such as text classification. However, the effectiveness of the data augmentation process can vary based on certain factors such as the specific LLM used, the selected parameters such as the temperature setting, and the downstream classifier applied.

This study provides preliminary evidence that open-source LLMs can improve the performance of text classifiers for small datasets in health care contexts when privacy or cost considerations prevent the use of closed-source third-party services such as those offered by OpenAI. These results pave the way for future research to further investigate and refine the use of LLMs in tasks like text classification, data augmentation, and other medical education and operational applications.

This research serves as an initial leap towards exploiting the promising capabilities of LLMs in medical applications while being mindful of privacy, ethical concerns, and constraints associated with this field. By establishing a proof-of-concept for the use of open-source LLMs in health care settings, this study opens avenues for broader exploration of LLMs’ potential to tackle numerous challenges faced by medical practitioners, educators, and administrators. Future research should expand on our work by exploring more complex datasets, experimenting with different hyperparameters for a wider variety of LLMs, and developing procedures to systematically craft and evaluate prompts to optimize model output.

This study contributes to a burgeoning field of research exploring applications of AI in health care and medical education. Our exploration of data augmentation using open-source LLMs presents potential pathways for improving processes such as incident reporting, resident evaluation, clinical vignette development, and other text-based processes relevant to medical education. This research will hopefully encourage additional exploration into the ethical and judicious application of LLMs and other AI technologies in health care.

As AI technologies continue to evolve and become more sophisticated, constant re-evaluation and updates to our methods will be essential. Therefore, active engagement from all stakeholders in the medical field, including frontline health care workers, researchers, educators, and policy makers, is crucial in making the most of these advancements for the betterment of patient care and safety.

## Abbreviations

AI: artificial intelligence
AUC: area under the receiver operating characteristic
DistilBERT: Distilled BERT
LLaMA: Large Language Model Meta AI
LLM: large language model
RETIPS: resilience engineering tool to improve patient safety
RoBERTa: Robustly Optimized BERT Pretraining Approach
